# Epidemiological profile of anterior cruciate ligament injuries in a tertiary referral trauma center of Nepal

**DOI:** 10.1186/s12891-022-05551-y

**Published:** 2022-06-21

**Authors:** Amit Joshi, Nagmani Singh, Bibek Basukala, Rohit Bista, Bibek Maharjan, Ishor Pradhan

**Affiliations:** AKB Center for Arthroscopy, Sports Injuries and Regenerative Medicine, B&B Hospital, Gwarko, Lalitpur, Nepal

**Keywords:** Anterior cruciate ligament, Road traffic accidents, Sports injury, Fall

## Abstract

**Background:**

Sports related injuries are the leading cause of Anterior Cruciate Ligament (ACL) tear in the Western world. Although professional and recreational sporting activities are increasing in Nepal, they are not as common and prevalent in comparison to western world. In contrast, Road Traffic Accident (RTA) is a very common cause of knee injuries in Nepal. Although there are some studies from Nepal mentioning Road Traffic Accidents (RTAs) as the most common cause of ACL injury, no specific studies have primarily investigated the epidemiological and demographic profile of ACL injured patients from this region. we aimed to understand the epidemiological and demographic profile of ACL injured patients and evaluate the mode of injury in a tertiary referral trauma center of Nepal.

**Methods:**

This was a retrospective descriptive study of a hospital cohort conducted from February 2018 to January 2020. Electronic details were retrieved, telephone interviews conducted and data analysis was done using descriptive analysis on the patients from the fore mentioned dates to complete demographic and epidemiological information.

**Results:**

A total of 237 patients were enrolled in this study. Among these, 120 patients (50.6%) fell into the age group of 15–30 years with a male to female ratio of 2.7:1. A RTA was the most common cause of ACL injury (38.8%), followed by sports-related injuries in 33.3% and falls in 16.5% of patients. The most common mode of RTA was a two-wheeler accident, and football was the most common sport causing ACL injuries. Sports injury was more common in patients below 30 years of age (OR = 3.5, 95% CI [2.2, 5.7]), whereas RTA was more common in patients above 30 years of age. Sports was the cause of ACL injury in 55.5% of students and RTAs was the commonest cause of ACL injury in office workers.

**Conclusion:**

Overall males were more frequently injured than females. Road traffic accidents were the most common cause of ACL injury in our subset of patients. Two-wheeler riders were the most commonly injured patients. Sport was the commonest cause of ACL injury in patients below 30 years, and RTA was common in patients above 30 years of age. Sports were the commonest cause of ACL injury in students, while RTA was the most common cause in office workers.

**Supplementary Information:**

The online version contains supplementary material available at 10.1186/s12891-022-05551-y.

## Background

The incidence of ACL injuries has increased over the past decade [1]. The possible cause of increased ACL injuries has been linked to increased participation in sports: at the professional and recreational levels [2, 3].

Several studies have labeled ACL injuries as sports injuries [4]. However, most of these studies and registries evaluating epidemiological factors have been reported from Europe, America, and other developed countries where sporting activities are part of their daily activity [4–6]. Although registries are the most valuable tool to analyze epidemiological parameters of an injury, the findings of a single registry should not be generalized to other populations throughout the world [7]. Nepal is known for its RTAs and challenging terrain. Trauma caused by RTAs is considered a more frequent cause of knee injury in Nepal and among RTAs; two-wheeler accidents were shown to be the most frequent in different studies [8, 9]. We believe that the mode of injuries in our context might differ from western and more developed societies because sporting activities are not part of our daily lives and RTAs are the most common cause of knee injuries in Nepal [8].

The goal of this study was to figure out the epidemiological and demographic profile of ACL injured patients and determine the mode of injury in a tertiary referral trauma center of Nepal. The findings of this study are expected to be critical in developing guidelines for the prevention and treatment of knee injuries and a better understanding of the condition.

## Methods

### Ethical approval and consent to participate

Ethical approval was obtained from the B&B hospital institutional review committee, Lalitpur Gwarko, (approval number 37). The electronic records (phone number and address) of patients who underwent ACL reconstruction (ACLR) at our center from February 2018 to January 2020 were retrieved from the record section. All the patients were contacted over the telephone by one of the authors (BM) and informed verbal consent to participate in this study was taken. Since this research required information about mode of injuries only and did not need examination of patients, an informed verbal consent was approved by IRC.

### Patient selection criteria

Patients not willing to participate and those who could not be contacted by telephone were excluded from the study. Revision ACLR, multi-ligament reconstructions and patients below 15 years of age were excluded from the study as this may have caused heterogenicity in subjects.

### Data extraction and synthesis

During the interview, the proforma specifically prepared for this study was completed. This proforma included details of patients and their epidemiological and demographic parameters. The proforma is attached as supplementary file (Additional file 1). The completed proforma was manually checked by the principal author (AJ) and approved for data entry.

The demographic data obtained on proforma were then sub-grouped based on age, sex, regions of residence (Himalayan, Hilly, and Terai), occupation, and level of education (uneducated, pre-school, school, and graduate). The mode of injury was recorded as RTA, Sports, Fall, and Others. The injuries that did not occur due to trauma, sports, or fall were categorized as others. Injuries occurring due to sports were also differentiated based on the type of sports. RTA was further subdivided into two-wheeler, four-wheeler, or pedestrian-related accidents.

### Statistical analysis

Data were entered manually into the IBM SPSS (IBM Corp. Released 2015. IBM SPSS Statistics for Windows, Version 23.0. Armonk, NY: IBM Corp.). The SPSS file is attached as supplementary file (Additional file 2). Shapiro-Wilk test was used to check the normality of age and mechanism of injury. Both of them were normally distributed. The Chi-square test was used to analyze the relationship between different groups.

**Fig. 1:**
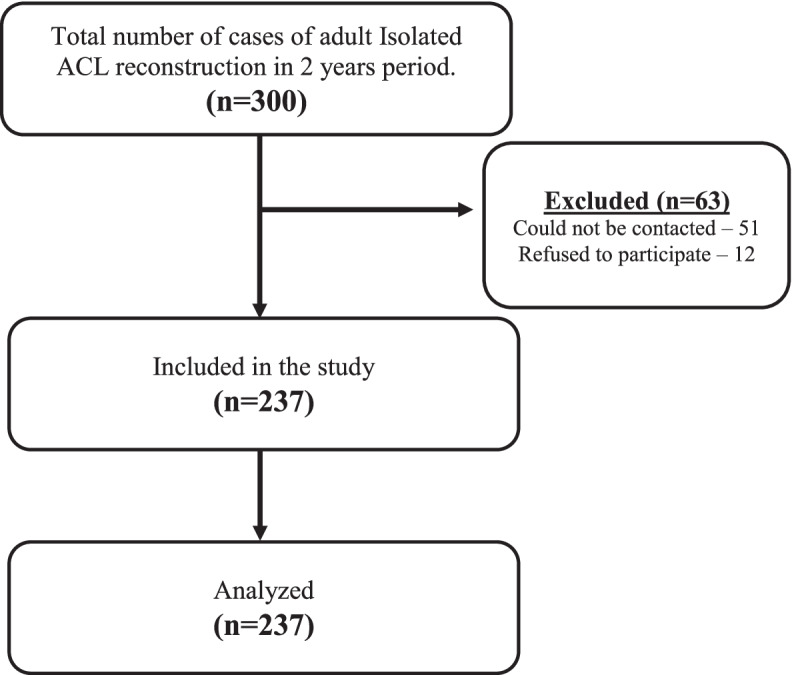
Flow diagram of subject selection

Electronic data of 300 cases of ACL reconstruction performed during the study period were retrieved (convenient sample). Out of the 300 patients, we were not able to establish contact with 51 patients and 12 patients declined to be part of the research. The patients were interviewed by telephone. A complete data set of the remaining 237 patients was used for final analysis (Fig. 1).

## Results

### Age and gender distribution

The mean age of our study population was 32.3 +/− 10.5 years, ranging from 15 to 67 years; male 29.8+/− 9.6 years, female 39.2+/− 10.2. The majority of patients (119 out of 237; 50.2%) fell in the age group of 15–30 years, followed by 29.5% in the 31–40 years group. Out of 237 patients who underwent isolated ACL reconstruction, 173 (73%) were male, and 64 (27%) were female, with a male to female ratio of 2.7:1 (Table 1).

**Table 1:** Demographic profile of patients

**Age Group**	
15–30 years	120 (50.6%)
31–45 years	89 (37.6%)
Above 45 years	28 (11.8%)
**Sex Distribution**	
Male	173 (73%)
Female	64 (27%)
**Geographical Distribution**	
Hilly region	143 (60.3%)
Terai region	90 (38%)
Himalayan	4 (1.7%)
**Occupation**	
Office worker	72 (30.4%)
Student	63 (26.6%)
Housewife	39 (16.5%)
Businessman	22 (9.3%)
Laborer	16 (6.8%)
Others	25 (10.5%)
**Education Level**	
School Level	113 (47.7%)
Graduation	79 (33.3%)
Pre-school	29 (12.2%)
Uneducated	16 (6.8%)

Sports injury was more common in patients below 30 years of age with odds ratio (OR) of 3.5, (OR = 3.5, 95% CI [2.2, 5.7]), whereas RTA was more common in patients above 30 years of age.

### Geographic distribution and occupations

Out of the 237 patients enrolled in this study, 143 (60.3%) were from the Hilly region, 90 (38%) from Terai and 4 (1.7%) from the Himalayan region (Table 1). A total of 72 (30.4%) patients were office workers, 63 (26.6%) students and 39 (16.5%) housewives (Table 1). The majority of patients 113 (47.7%) have completed their school level education, followed by 79 (33.3%) who have completed graduation level education (Table 1).

The OR of having sports injuries in Terai was 0.6 (OR = 0.6, 95%CI [0.4, 1.0]) compared to 1.2 (OR = 1.2, 95%CI [1.0, 1.4]) in Hilly region. Similarly, the OR of RTA in Terai was 0.7 (OR = 0.7, 95%CI [0.5–1.0]) compared to 1.1 (OR = 1.1, 95%CI [0.9–1.4]) in Hilly region.

### Mode of injury

**Fig. 2:**
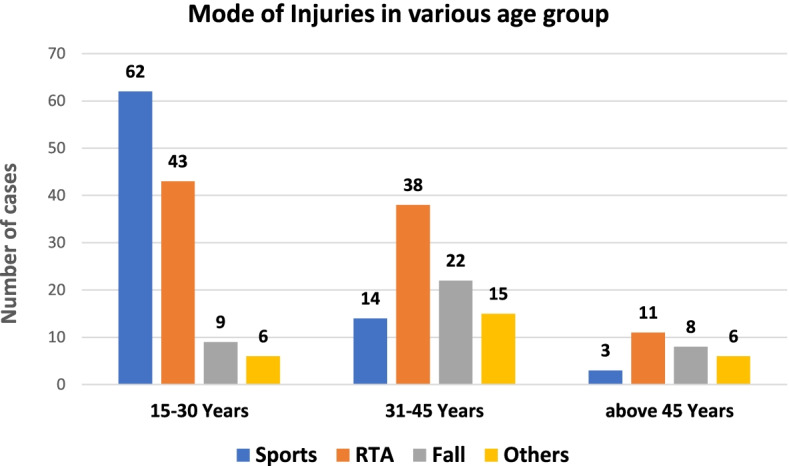
Mode of injury in the various age group

Out of 237 patients, 92 (38.8%) had sustained knee injuries because of RTA, followed by sports-related in 79 (33.3%) and falls in 39 (16.5%) (Fig. 2).

Among 92 patients who injured their ACL because of RTA, 71 (77.2%) sustained injuries due to two-wheeler accidents and only 8 (8.7%) sustained an injury because of a four-wheeler accident. Among the 13 pedestrians who sustained an ACL injury, 9 were hit by a two-wheeler. Football was the most common sport resulting in 83% (59 out of 79) of sports-related injuries, followed by Basketball and Badminton (Table 2).

**Table 2:** Subdivision of RTA and sports injury

**RTA (*n*** **= 92)**	
2-wheeler (*n* = 71)	
Rider 66 (93%)	
Pillion 5 (7%)	
4-wheeler (*n* = 8)	
Driver - 1 (12.5%)	
Occupant - 7 (87.5%)	
Pedestrian (*n* = 13)	
Hit by 2-wheeler 9 (69.2%)	
Hit by 4-wheeler 4 (30.8%)	
**Sports (*n*** **= 79)**	
Football 59 (83%)	
Basketball 9 (12.7%)	
Badminton 4 (5.6%)	
Cricket 4 (5.6%)	
Volleyball 3 (4.1%)	

### Mode of injuries in various subgroups

Among 92 patients who sustained ACL injury because of RTA, 68 (74%) were male and 24 (26%) females. Similarly, among 79 patients who sustained ACL injuries due to sporting activities, 76 (96.2%) were male and only 3 (3.8%) were female. Females were more frequently involved in ACL injuries caused by falls and others. The OR of sports related injuries for male was 1.5 (OR = 1.5, 95%CI [1.3, 1.7]) whereas OR of sports-related injuries for female was 0 (OR = 0, 95%CI. [0, 0.3]).

**Fig. 3:**
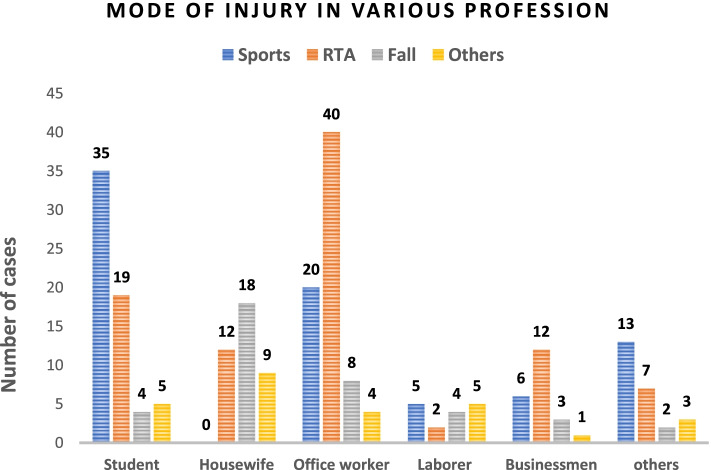
Mode of injury based on the profession of patients

Sports was the most frequent cause of ACL injury in the age group of 15–30 years, whereas RTA was the most common cause of ACL injuries in patients above 30 years (Fig. 3). The OR of sports-related injuries for patients below 30 years of age was 2.1 (OR = 2.1, 95%CI [1.6, 2.7]) whereas for patients above 30 years of age was only 0.3 (OR = 0.3, 95%CI [0.2, 0.5]).

The majority of students sustained ACL injuries because of sports-related causes, whereas fall and RTA was the cause of ACL injury in housewives. The most typical mode of injury was RTA for office workers and businessmen (Fig. 3).

Only 49.3% of patients with sports-related injuries underwent surgery within three months compared to 72.8% of patients with ACL injuries caused by RTA (Table 3). OR of delay in surgery (more than 4 weeks) with sports related ACL injuries was 1.3 (OR = 1.3, 95%CI [1.1, 1.6]).

**Table 3:** Surgical delay based on the mode of injuries

Mode of injury	Surgery delay					
	< 1 month	1–3 months	3–6 months	6–12 months	> 12 months	Total
RTA	53(57.6%)	14(15.2%)	14(15.2%)	2(2.2%)	9(9.8%)	92
Sports	16(20.2%)	23(29.1%)	15(19%)	2(2.5%)	23(29.1%)	79
Fall	9(23%)	12(30.7%)	6(15.4%)	2(6.9%)	10(25.6%)	39
Others	3(11.1%)	9(33.3%)	4(14.8%)	1(3.7%)	10(37%)	27
Total	81	58	39	7	52	237

## Discussion

The most important finding of this research was that RTA was the most common cause of ACL injury (38.8%, 92/237) followed by sports-related injuries 33.3% (79/237) and fall was the third leading cause of ACL injury, which accounted for 16.5% (39/237) of cases.

Although ACL injuries are considered sports injuries, sporting activities are still limited to a small interested specific population in our country [10]. Recreational and professional sporting activities are on the rise in our country but they are not widespread and are not the most common cause of knee injuries. Non-sports-related causes (RTA, fall, and others) constituted most ACL injuries (66.7%) in our study. Similar to our result, previous research [11] evaluating meniscal injury in patients with an ACL tear and another paper from Nepal [12] evaluating the outcome of single-bundle ACLR also mentioned RTA being the most common cause of ACL injury. However, those studies did not elaborate on the several epidemiological aspects of ACL injury and focused primarily on the incidence of meniscal injury. In this study, sports was the most common cause of ACL injury in patients below 30 years of age. In contrast, RTA was the most common cause of injury in patients above 30 years. The reason for this outcome could be because sports activities are popular among students who are usually below 30 years; and patients above 30 were office workers.

In contrast to our findings, a study from Singapore found that only 17.6% of their cases had sustained ACL injury because of non-sports activity [13]. The Norwegian registry reports non-sporting activities contributing to 24% of cases, whereas the Kaiser Permanente registry reports it to be 29.5% [14]. As per Kaiser Permanente and Norwegian registries, RTA only constituted 4.2 and 1.1% of total ACL injuries, respectively [14]. Meanwhile, Luxembourg, UK, and Swedish registries reported it to be 1, 1.1, and 4.4%, respectively [4]. This is in sharp contrast to our study, which had a significantly higher percentage of cases occurring due to RTAs (38.8%). Moreover, accidents involving two-wheelers constituted 86.9% of cases of ACL injuries. Among these, 77.1% of the cases were riding on the two-wheeler, and drivers were injured more than the pillion riders. Since the most usual mode of transportation in Nepal is on two-wheelers, it is possible that two-wheeler accidents are more common statistically [15].

Also, several studies published from all over the world have mentioned sports injury as the most common cause of ACL tear [4, 16–20]. According to registries, Football (Soccer) has been reported as the most common sports activity causing ACL injuries. In the Singaporean population, sports activities resulted in 83.4% of the injuries, out of which the most common sport was football [13]. Although sports-related activities were the second most common cause of ACL injury in our study, soccer was the most common sport (83%) in our study, followed by basketball, badminton, cricket, and volleyball. Football is the most popular sport amongst young Nepalese; hence is the leading cause of ACL injuries in this age group.

In our study, most injuries occurred in the 15–30 years age group, and 73% of patients were male, with a male to female ratio of 2.7:1. The male to female ratio in the RTA group was 2.8:1, whereas it was 25:1 in the sports group. In contrast, females were more in Fall and other groups. This disparity may be because fewer women are involved in sporting activities compared to their male counterparts in Nepal. Additionally, men are more prone to injuries because of being mobile and frequenting places while women are comparatively occupied within the boundaries of the house. Our findings are similar to that of other published registry cohorts in which patients were mostly male and < 30 years of age [4]. Luxembourg and UK registries [4, 21, 22] had similar distribution like ours, with 71.7 and 72.4% male cases, respectively. Singaporean cohort had an average age of 29.4 +/− 8.69 years, with a relatively higher number of male patients than ours (83.9%) [13]. The mean age of females (39.27+/− 10.2 years) was higher than those of male patients (29.8+/− 9.6 years) in our study. In contrast, the Swedish registry [23] mentioned that the average age of men at surgery is higher than that of women. Luxembourg registry [21] (men: 28 ± 10; women: 32 ± 12, *P* < 0.01) reported findings similar to our study but with a relatively lower average age of females. The possible explanation for women having an injury at higher age is that they are less involved in sports, which in turn results in injury at a younger age, and the majority of the injury in this group are due to RTA and falls, which occur predominantly in the older age group.

Based on the profession of the patients, RTA (43.47%) was the most common injury in office worker, followed by sports injuries and falls. The RTA is the most common mode because office workers mostly travel by motorcycle and are more prone to accidents. In contrast, ACL injuries in housewives were mainly due to falls and twisting, and there was no sports-related injury in this group. The housewives in Nepal are presumably not very active in sports or athletics due to socio-cultural barriers, so there are no sports-related ACL tears in this group. Sports-related ACL injuries are the most common mode in students, followed by RTA. In Nepal, students partake in various sporting activities during their school and college level of study and the sporting activities start waning after that time period. Two-wheelers are very popular among student for traveling around and could be a reason why RTA is the second most common mode of injury in this age group.

We also evaluated the influence of geographical distribution on the occurrence of ACL injuries. It was found that the majority of injuries occurred in people residing in Hilly areas (60.3%), followed by people from Terai (38%), and only 4 cases were reported from the Himalayan region. Sports activities were the most common mode of injury in the Hilly region followed by RTA and falls. RTAs caused most cases in Terai, followed by sports activities. Football is frequently played in Hilly regions of Nepal, and two-wheeler ownership is higher in the Terai region [15]. This explains why sports-related injuries are more common in the Hilly region and RTAs in Terai. Also, Football is not very popular in Terai, which might be the possible reason for fewer sports-related ACL injuries comparatively.

Patients in our study had an average delay of 296.8+/− 696.8 days (Range 1 day to 14 years) from injury to surgery. The median delay in the MOON cohort [24] was 2.4 months, and an average of more than six months delay was seen in Scandinavian cohorts [4]. We also calculated the average delay from injury to ACLR based on the mode of injury in our context. In our study, 56.7% of RTAs were operated on within a month from the date of injury, while the sports injury group had a more significant delay in seeking ACLR (mean delay-11.79 months). This delay can be explained because most RTA is reported early to hospitals for receiving treatments since the medical expenses are covered by a third party causing the accident. On the other hand, sports injury cases are reported later to hospitals since treatment expenses might be a constraining factor for the delay in surgery. Analysis of outcome based on presentation delay would have been interesting, but this was beyond the scope of this study.

### Limitations

There were several limitations of this study and the major one being the study focusing cases from a single center which did not portray a complete comprehensive profile of the injury of the country. The study also excluded non-operatively managed cases. Other limitations include the retrospective nature of study, a small sample size, a short sample period of two years and a lack of power analysis. Furthermore, 51 cases (17%) were excluded due to lack of verifiable data from patients which might have affected power of analysis and generalizability of the findings. A multi-center study consisting of larger sample size and duration from different parts of the country would have been ideal but we believe that this study represents a significant proportion of the patients presenting at tertiary centers of Nepal as this is one of the largest series from this part of the world where registries are not existing.

## Conclusion

The most common mode of ACL injury in a tertiary referral trauma center of Nepal was from RTAs - mostly two wheeled vehicles- followed by sporting activities and lastly from falling down. Sports injury and RTA were the most common mode of injury in patients under 30 and above 30 years of age respectively. Comparatively, patients that had ACL torn by RTAs underwent surgery sooner than patients who had the same condition through sports injuries.

## Supplementary Information

**Additional file 1.** Proforma of the research. This file contains all the epidemiological and demographic parametes which were asked to the patients during interview.

**Additional file 2.** SPSS data of epidemiology research. This file contains raw data of all the patients, which was used for analysis.

## Data Availability

All data generated or analyzed during this study are included in this published article as supplementary information files.

## Abbreviations

ACL: Anterior Cruciate Ligament
ACLR: Anterior Cruciate Ligament Reconstruction
RTA: Road Traffic Accident
RTAs: Road Traffic Accidents
B&B Hospital: Baidhya and Banskota Hospital
OR: Odds Ratio
CI: Confidence Interval
BM: Bibek Maharjan
AJ: Amit Joshi
IBM SPSS: International Business Machines Statistical Package for the Social Sciences
UK: United Kingdom
MOON: Multicenter Orthopedic Outcomes Network
