# Supplementary material for: Epidemiological profile of anterior cruciate ligament injuries in a tertiary referral trauma center of Nepal
Source: BMC Musculoskelet Disord. 2022 Jun 21;23:595. doi: 10.1186/s12891-022-05551-y (PMC9210789; doi:10.1186/s12891-022-05551-y)
Supplement: Supplementary file 1 — Additional file 1. Proforma of the research. This file contains all the epidemiological and demographic parametes which were asked to the patients during interview. [file 12891_2022_5551_MOESM1_ESM.pdf]

Research No. 167
IRC approval No. 37

Note: This proforma has to be filled only after informed verbal consent from patient.

Proforma for epidemiology of meniscal tear in ACL deficiency patient

1.	Name	
2.	Contact Number	
3.	Age in years	
4.	Sex	Male ☐ Female. ☐ Other ☐
5.	Region	Himalayan ☐ Hilly ☐ Terai ☐
6.	Occupation	Office worker. ☐ Student ☐<br>Housewife ☐ Businessman ☐<br>Laborer ☐ Others ☐
7.	Education	Uneducated. ☐ Preschool ☐<br>School. ☐ Graduate ☐
8.	Side	Right ☐<br>Left ☐<br>Bilateral ☐
9.	Mechanism of injury	1) Sports ……………………<br>2) RTA<br>a) two-wheeler<br>☐Pillion<br>☐Rider<br>b) four-wheeler<br>3) c) Pedestrian<br>☐two-wheeler<br>☐four-wheeler<br>4) Fall<br>5) Others<br>………………………………………<br>…………………<br>………………………………………<br>…………………………….
10.	Surgery delay from time of injury (in months)	

Signature of data collector
Dr. Bibek Maharjan

Signature of data verifier
Dr. Amit Joshi
